# On the Influence of Welding Parameters and Their Interdependence During Robotic Continuous Ultrasonic Welding of Carbon Fibre Reinforced Thermoplastics

**DOI:** 10.3390/ma17215282

**Published:** 2024-10-30

**Authors:** Filipp Köhler, Irene Fernandez Villegas, Clemens Dransfeld, Axel Herrmann

**Affiliations:** 1 Composite Technology Center Stade, Airbus Straße 1, 21864 Stade, Germany; 2 Aerospace Structures and Materials, Faculty of Aerospace Engineering, Delft University of Technology, Kluyverweg 1, 2629 Delft, The Netherlands; 3 Faculty of Production Engineering, University of Bremen, Bibliothekstraße 1, 28359 Bremen, Germany

**Keywords:** thermoplastic composites, fusion bonding, energy director, unidirectional fibres

## Abstract

Ultrasonic welding of fibre-reinforced thermoplastics is a joining technology with high potential for short welding times and low energy consumption. While the majority of the current studies on continuous ultrasonic welding have so far focused on woven reinforcements, unidirectional materials are preferred for highly loaded aerospace components due to their better mechanical performance. Therefore, this paper investigates the influence and interdependence of the welding speed, amplitude, and energy director thickness on the weld quality of adherends made of unidirectional composites. The quality of the welded joints is assessed by a single-lap shear strength and fracture surface analysis complemented by the microscopic analysis of cross-sections and comparison to a co-consolidated reference. The results showed that the welding process is highly affected by changing welding speeds for a given amplitude. Furthermore, while lower amplitudes lead to significant scatter in the welding quality, higher amplitudes result in increased heating rates and a fully molten energy director even for high welding speeds. Nevertheless, insufficient consolidation at high welding speeds results in porosity in the weld line. Finally, it was observed that thicker, and therefore more compliant, energy directors lead to more uniform melting of the energy director and less deviation in the weld quality for a wider range of welding speeds.

## 1. Introduction

Fibre-reinforced thermoplastic composites offer unique potential for the manufacturing and assembly of aerospace components. Joining technologies play a key role in the use of those potentials. Ultrasonic welding is one of those joining technologies, with a high potential for short welding times and low energy consumption. During ultrasonic welding, low-amplitude and high-frequency vibrations are applied transversally to the welding surface through a sonotrode [1,2]. Heat generation occurs at the weld interface due to frictional and viscoelastic heating of the polymer [3]. Due to its lower stiffness compared to the fibre-reinforced adherends and, therefore, higher cyclic strains, heat is generated mostly in the so-called energy director (ED) [4]. It usually consists of a thin neat-polymer element placed between the adherends. It can be a triangular protrusion moulded on one of the adherends [5], a thin polymer film [6], or a woven polymer mesh [7]. Ultrasonic welding can be a static or continuous process. The main difference between the two approaches is that, during the continuous process, the sonotrode and the adherends move relative to each other; hence, the heating, melting, cooling, and consolidation phases are not only temporally but also spatially separated in in the continuous process.

In the past, a large amount of focus was devoted to understanding the static welding process and the influence of the different welding parameters, such as the energy director type, the welding force, and the vibration amplitude. For instance, Palardy et al. [8] conducted a deep analysis on the influence of the thickness of energy directors, in the shape of thin polymer films, on the static welding process. According to their study, the use of very thin (0.06 mm) energy directors causes simultaneous heating and melting of the energy director and of the adherends, which makes process control and the prevention of overheating challenging. This is not the case when thicker (0.25 and 0.50 mm, in that study) energy directors are used; however, they lead to relatively longer welding times owing to the lower cyclic strain in the ED [8].

The vibration amplitude and the welding force are important welding parameters as well. Both parameters influence the welding process and the final weld quality, and affect the way the energy director melts. Both were studied in previous research [9,10,11]. Usually, the melting of the energy director relies on a combination of the nucleation of hot spots and their growth. Villegas [9] found that the growth of hot spots is constrained when using low amplitudes, as the heating rates are decreased and heat is transferred in the comparatively cold composites. On the other hand, the nucleation of new hots spots is affected mainly by the welding force, as a high welding force increases the intimate contact between the adherends and the energy director, leading to more nucleation sites. Static ultrasonic welding also allows for in situ monitoring by using different process data. Villegas proposed a relationship between the process data and lap shear strength [9]. According to her findings, the vertical displacement of the sonotrode can be used to control the process and achieve high-strength welds. The vertical displacement is the result of local squeeze flow of the molten energy director. 

Regarding the continuous process, Senders et al. [12] developed one of the first approaches to continuous ultrasonic welding by continuously translating a static ultrasonic welding end effector along the line to be welded. They also used a very thin film energy director in the range of 0.08 mm thickness to prevent excessive thickness differences between the welded and un-welded overlap as the sonotrode moves along. The result of using such a thin energy director is simultaneous heating of the energy director and the adherends without or with only little squeeze flow. In their approach, referred to as zero-flow continuous ultrasonic welding, they were able to produce high-strength joints using PPS fabric materials but encountered porosity and un-welded areas. To improve the weld uniformity, Jongbloed et al. [7] investigated the effect of the compliance of the energy directors on the welding process. Apart from films with different thicknesses and, therefore, compliances, they introduced a polymer woven mesh energy director which improved the weld uniformity significantly. They attributed this improvement to the more uniform contact between the energy director and the adherend compared to the relatively stiffer films.

However, within both previously mentioned studies, voids were encountered when analysing the fracture surfaces of the welded joints after mechanical testing. They attributed this to insufficient consolidation pressure, which could be solved by using a consolidation device following the sonotrode.

The majority of the studies on continuous ultrasonic welding have so far focused on fabric material. Unidirectional materials are, however, often preferred for highly loaded aerospace components due to the absence of crimp, which increases the strength and stiffness. The use of unidirectional material instead of fabrics could increase the applicability of ultrasonic welding to highly loaded parts. Transferring and adapting our knowledge from fabric materials to unidirectional materials requires specific attention, as unidirectional materials impose different requirements on the welding process, which was shown within our work on industrialisation aspects of large thermoplastic aerospace structures [13]. To allow application of continuous ultrasonic welding on actual components, the influence of different welding parameters has to be understood.

We also argued in our presented roadmap that a robot could be a flexible solution when it comes to different applications of ultrasonic welding and could therefore extend the applicability of the process [14]. The general feasibility of a robotic solution was already shown by Engelschall et al. [11].

This paper investigates the influence and interdependence of the welding speed, amplitude, and energy director thickness on the weld quality of adherends made of unidirectional composites and welded by robotic continuous ultrasonic welding. The quality of the welded joints is assessed by single-lap shear strength and fracture surface analysis complemented by the microscopic analysis of cross-sections and comparison to a co-consolidated reference.

## 2. Material and Methods

### 2.1. Material

The material used in this study was Toray Cetex^®^ TC1225 unidirectional prepreg tape from Toray Advanced Composites (TAC, Nijverdal, The Netherlands). The material was delivered as already press-consolidated laminates by the supplier. The laminates were composed of 12 plies with the stacking sequence (0/135/90/135/45/135)_s_. They were inspected by ultrasonic C-scan for voids and delaminations. Ultrasonic welding specimens (250 mm × 101.6 mm) were extracted from areas without defects by water jet cutting, where the 0° top layer was oriented in the 101.6 mm direction. Samples were degreased and cleaned with acetone prior to welding. Samples were not dried before welding. The material has a melting temperature (Tm) of 305 °C and a glass transition temperature (T_g_) of 147 °C, and was processed within the process boundaries of 340–385 °C to manufacture the laminates.

Two energy directors were used in this study. Both of them were made of Victrex LMPAEK™ Polymer film (Victrex, Hofheim, Germany), a similar polymer as used in the prepreg material, and were delivered as discontinuous films. The polymer film has a Tg of 152 °C and Tm of 303 °C, which is very similar to the consolidated laminates. One type of ED, referred to as the “thin energy director”, had an areal weight of 160 g/m^2^. The other one, referred to as the “thick energy director”, had an areal weight of 400 g/m^2^ and was 2.5 times thicker than the thin one. The energy directors were cut into 290 × 30 mm^2^ pieces to be larger than the intended welding area and were secured by adhesive tape to the lower adherend. The thin energy director was chosen based on previous research where a similar energy director was used [8]. To study the influence of the energy director thickness, an energy director with a significant thickness difference was chosen.

### 2.2. Continuous Ultrasonic Welding

**Figure 1:**
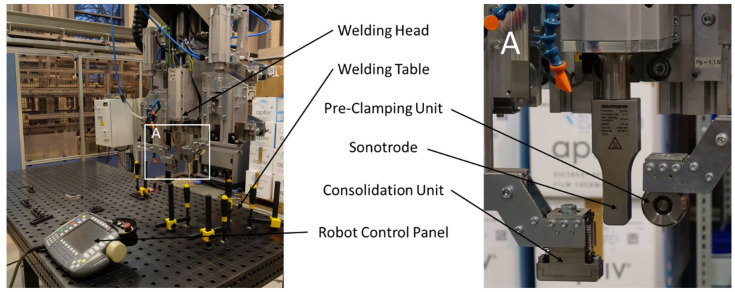
Welding setup used throughout the study. The welding head including the ultrasonic welder, the consolidation unit, and the pre-clamping unit, which was mounted to a Kuka robot.

A 20 kHz micro-processor-controlled ultrasonic welder from Herrmann Ultraschalltechnik, Karlsbad, Germany, was used to weld specimens in a single-lap shear configuration with an overlap of 12.7 mm. In the continuous ultrasonic welding setup used in this study, a welding head (Figure 1) provided with a rectangular sonotrode with dimensions of 14.9 × 27 mm^2^ was continuously moved to create a weld seam of 250 × 12.7 mm^2^. The welding head was mounted onto a Kuka KR 125 (Kuka, Augsburg, Germany) robot equipped with a KR C2 controller. The robot was allowed to move the welding head with a maximum speed of ca. 1000 mm/s along the intended weld seam, although the welding speeds used in the present study ranged between 4 mm/s and 36 mm/s.

The ultrasonic welder had a maximum power output of 6.2 kW and automatically adjusted the power to keep the vibration amplitude constant. The maximum peak-to-peak amplitude that could be set was 90 µm. Here, vibration amplitudes amounting to 50%, 75%, and 100% of the maximum available amplitude were used. Static welding pre-trials were performed to define the lowest possible amplitude. Amplitudes below 50% of the maximum amplitude could not achieve melting of the energy director even when welding times of 8 s or longer were used.

The study was conducted in three major steps. Within the first step the influence of the welding speed was investigated by applying different welding speeds using a fixed amplitude and energy director. In the second step the influence of the welding speed using higher and lower vibration amplitudes was investigated. The energy director was again kept fixed. In the last step of the research the influence of the welding speed when using different energy director thicknesses was investigated. In this case again the vibration amplitude was kept to a fixed value. Table 1 summarises the applied process parameters and their combinations.

The welding force F_w_ was set to 500 N, corresponding to a welding pressure of 2.64 MPa for the contact area of the sonotrode. To allow for sufficient consolidation of the welded joint, a consolidation unit with dimensions 60 × 27 mm^2^ followed the sonotrode at a distance of 10 mm. The consolidation unit was mounted as close as possible to the sonotrode but contact between the consolidation device and the sonotrode had to be avoided to prevent damage to either one. To counterbalance the consolidation force F_c_ and avoid bending moments introduced into the robot axes, a pre-clamping roll was mounted ahead of the sonotrode (see Figure 1). The distance of the pre-clamping roll to the sonotrode and the applied force F_p_ were the same as for the consolidation unit. The consolidation force was set to 600 N, which corresponds to a consolidation pressure of 0.79 MPa. The consolidation force F_c_ = F_p_ was limited by the welding force, the maximum payload of the robot (125 kg), and the weight of the welding head to avoid dangerous weld conditions. The sum of all applied forces must not exceed 1700 N, including the welding force, the consolidation force, and the force applied by the pre-clamping roll. The consolidation time *t_c_* was determined by the welding speed *v_w_* and the length of the consolidation unit *l_c_* and could be calculated for each set of weld parameters according to the following equation.

(1) $$t_{c}=\frac{l_{c}}{v_{w}}$$

**Figure 2:**
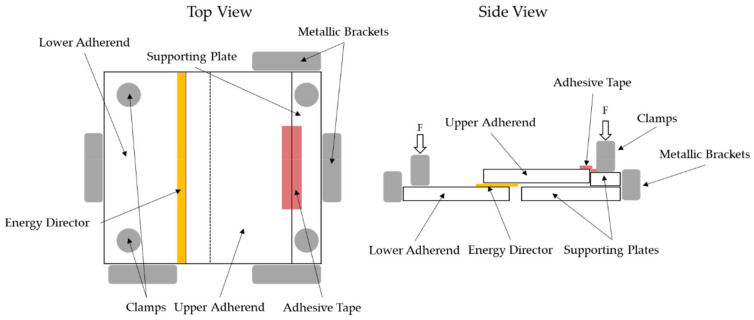
Schematics of custom-made welding jig which prevents the specimens from moving during the welding operation.

The specimens were held in place by a custom-made jig as shown in Figure 2. The jig prevented the specimens from moving during the welding process and allowed for free access to the weld area. To avoid misalignment of the two adherends, the upper adherend was not rigidly clamped to allow for vertical displacement during the welding process. Instead, the upper adherend was only restricted by the metallic brackets and the applied adhesive tape shown in Figure 2.

### 2.3. Testing and Evaluation

**Figure 3:**
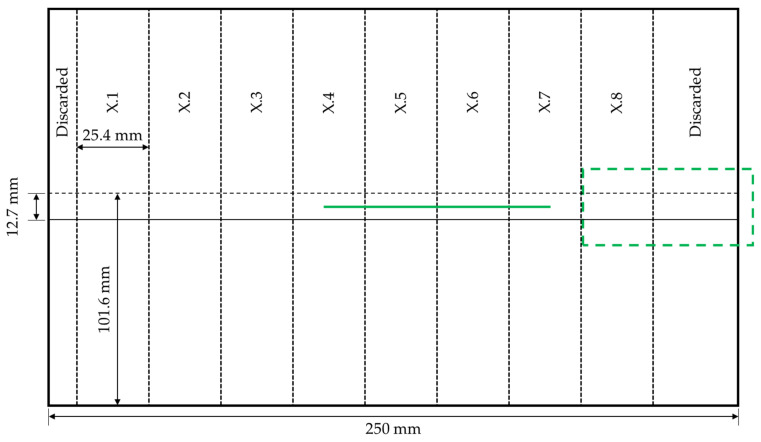
Schematic of the cutting scheme for mechanical testing. The green solid line indicates the position of the microscopic cross-section and the dashed rectangular line the area of longer consolidation time.

After the continuous ultrasonic welding process, the adherends were cut into 25.4 mm-wide single-lap shear samples using a Proth water-cooled grinding machine. The cutting scheme is shown in Figure 3. Areas at the beginning and at the end of the weld area were discarded as the welding and consolidation conditions in these areas differed from those in the rest of the weld area. The width of the discarded area at the end of the weld line was chosen to allow the extraction of sample X.8, as it was the only sample with a longer consolidation time.

Mechanical testing was performed following ASTM D1002 using a Zwick/Roell 250 kN universal testing machine (Zwick/Roell, Ulm, Germany) with a cross-head speed of 1.3 mm/min. The hydraulic grips of the testing machine were offset to ensure parallelism between the weld line and the load path. The lap shear strength was calculated based on the maximum load divided by the total weld overlap (12.7 mm × 25.4 mm). For the calculation of the average lap shear strength, only the samples X.3–X.7 were considered (Figure 3), as samples X.1 and X.2, as well as X.8, were subject to different welding and consolidation conditions. Samples X.1 and X.2 were suspected to have been affected by the start of the welding process, as the start of the vibrations and the start of the continuous movement happened simultaneously and the welding amplitude was required to ramp up in the beginning. Furthermore, the consolidation unit was applied once full contact with the surface of the upper adherend could be assured. At this point, the sonotrode was already in the centre of sample X.2. For X.8, the welding conditions were comparable to the samples X.3–X.7 but the sample was subject to a longer consolidation time, as the consolidation unit remained at the location of the X.8 sample for a minimum of 8 s before it was retrieved. Sample X.8 was hence considered separately within this study, while samples X.1 and X.2 were not considered further.

The calculated lap shear strengths were compared to a consolidated reference laminate, made of 24 plies, and are given as percentages of the average lap shear strength achieved by consolidation. The consolidated reference was manufactured using hand layup and subsequent press consolidation, and following the supplier’s processing guidelines. A consolidation pressure of 10 bar and a dwell time of 30 min were applied at ca. 365 °C. A cooling rate of <10 °C/min was applied to allow for full crystallisation of the polymer. Afterwards, samples were prepared similarly to the welded samples. Slots were introduced to achieve an effective testing area of 12.7 mm × 25.4 mm, equal to the ultrasonically welded samples.

The fracture surfaces were analysed with the naked eye. Specimens were cut along the weld line (Figure 3) to perform cross-sectional microscopy. Samples for microscopy were prepared by cutting samples with a precision saw, embedding them in epoxy resin, and grinding and polishing them using several steps. Microscopy was performed by using a Leica reflected-light microscope with a magnification of 50×.

## 3. Results

### 3.1. Baseline Welding Parameters

**Figure 4:**
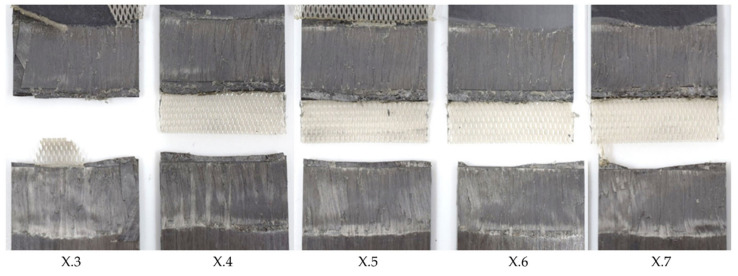
Fracture surface of samples welded with 75% amplitude (samples X.3–X.7 from left to right), 10 mm/s welding speed, and the thin energy director. The fracture surface shows no sign of fibre squeeze out or porosity.

During a series of pre-trials, baseline welding parameters were defined as a starting point for this study. An energy director with an areal weight of 160 g/m^2^, a welding speed of 12 mm/s, and vibration amplitude of 75% of the maximum amplitude were found to deliver a satisfactory weld quality. In this case, satisfactory weld quality meant that the fracture surfaces showed no signs of polymer degradation such as discoloured areas, significant fibre squeeze out, or porosity in the weld interface (see e.g., Figure 4). The average lap shear strength of the satisfactory samples from the pretrials was found to be 96% (±1%) of the consolidated reference.

**Figure 5:**
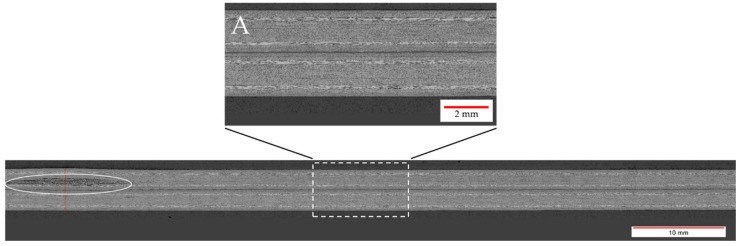
Microscopic cross-section of a sample welded with 75% amplitude at 10 mm/s using the thin energy director. Detail A shows a close-up of the weld line, showing good welding quality. The white circled area shows a porosity within the upper adherend most likely attributable to a local hot spot.

Additionally, the microscopic longitudinal cross-section (as defined in Figure 3) along the weld area was analysed. Figure 5 shows that the weld line itself does not show any visible defects and also appears very uniform in its thickness. Nevertheless, towards the left edge of the microscopic image, an area with significant voids within the upper adherend is visible. Although the root causes cannot be fully explained, it is most likely that the defect is linked to a local hot spot caused by variations in the thickness, the energy director, or prior defects in the adherends. These defects were already visible through the visual inspection of the top adherend before microscopic cross-section or mechanical testing were performed. It has to be noted that this defect had no impact on the lap shear strength, as the lap shear strength respective sample (X.4) is not significantly different from the average lap shear strength and also the respective fracture surface does not show any specific features (Figure 4). Such local effects were occasionally seen throughout the study, but no systematic root cause could be identified.

### 3.2. Welding Speed

**Figure 6:**
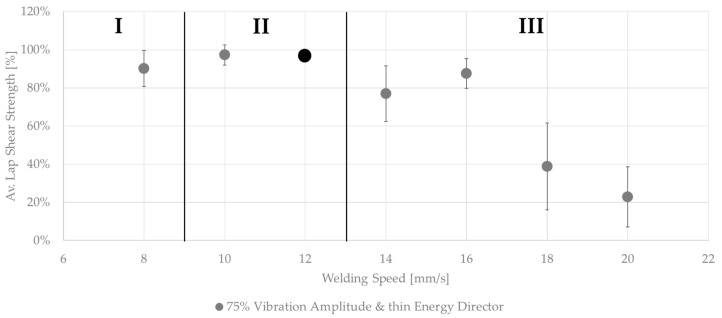
Average lap shear strength of samples welded with 75% amplitude using the thin energy director at different welding speeds. The results are sorted into three categories depending on the weld quality. The black dot represents the initial welding parameters.

**Figure 7:**
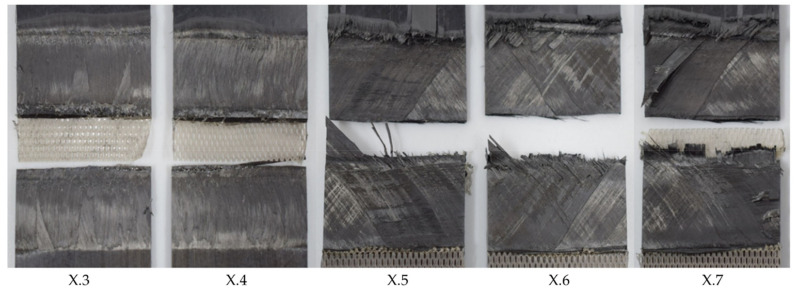
Fracture surface of sample welded with 75% amplitude and 8 mm/s welding speed using the thin energy director (samples X.3–X.7 from left to right). The fracture surface shows fibre squeeze out at failure partially within the 135° ply.

**Figure 8:**
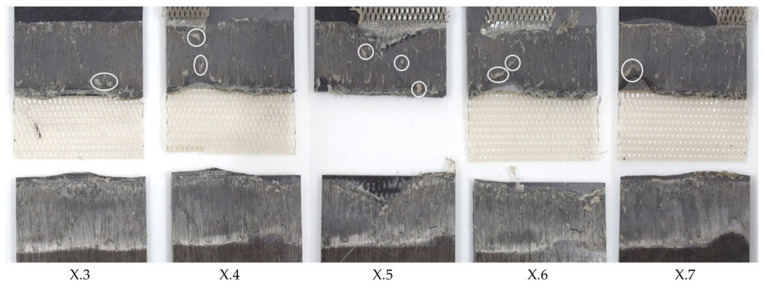
Fracture surface of sample welded with 75% amplitude and 16 mm/s welding speed using the thin energy director (samples X.3–X.7 from left to right). The fracture surface shows un-welded energy director mostly at the transversal edges. The white circles indicate resin-rich areas.

**Figure 9:**
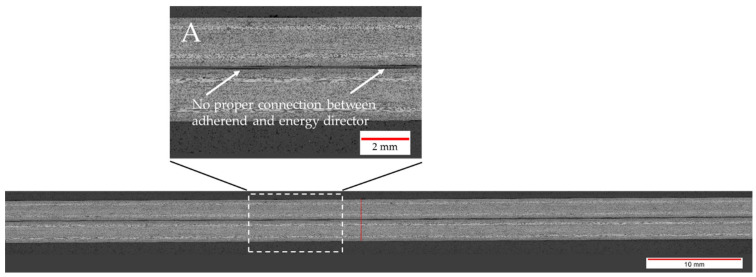
Microscopic cross-section of a sample welded with 75% amplitude at 16 mm/s using the thin energy director. Detail A shows areas of improper connection between the energy director and the adherends.

Figure 6 shows the effect on the average lap shear when using welding speeds that were higher or lower than the initial welding speed of 12 mm/s. The results could be divided into three categories. Welds that fell into category II (10–12 mm/s) showed the same or a similar weld quality as the baseline welding parameters, while, for welds within category I (below 10 mm/s), a slight drop in the average lap shear strength was observed. Additionally, those welds showed different fracture surfaces, as can be seen in Figure 7. Significant fibre squeeze out at the transversal edges was observed, which finally led to failure within the 135° layer instead of the top 0° ply as was the case for the initial welding parameters. Finally, welds within category III (above 12 mm/s) showed a steep decrease in lap shear strength and significant scatter. Figure 8 shows the fracture surfaces of a sample welded at 16 mm/s. This sample was a typical example for a weld that fell into category III. The fracture surfaces revealed un-welded energy director, especially at the transversal edges and resin-rich areas, indicated by the white circles within Figure 8. The corresponding microscopic cross-section (Figure 9) confirmed these observations, as it showed areas with a lack of intimate contact between the energy director and the adherends.

### 3.3. Amplitude

**Figure 10:**
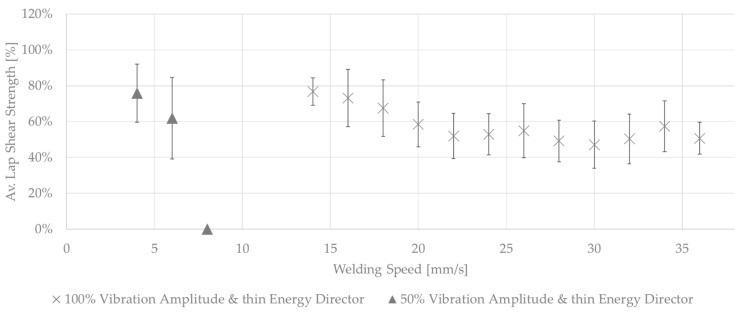
Average lap shear strength of samples welded with 100% and 50% amplitudes using the thin energy director at different welding speeds.

**Figure 11:**
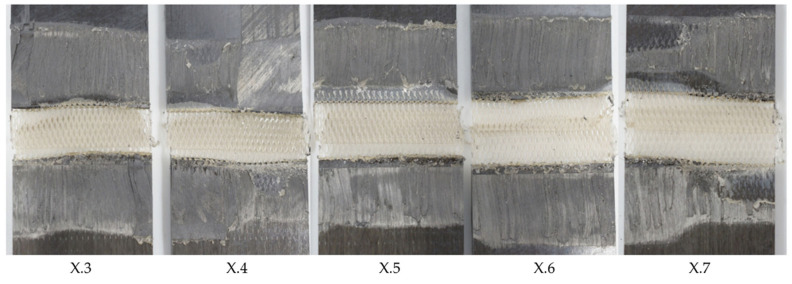
Fracture surface of sample welded with 50% amplitude and 4 mm/s welding speed using the thin energy director (samples X.3–X.7 from left to right). The fracture surface shows varying amounts of un-welded energy director from sample to sample.

Figure 10 shows the average lap strengths for samples welded at different welding speeds and at higher or lower vibration amplitudes than the baseline amplitude. The welds performed with 50% amplitude were characterized by a large scatter in weld quality, a rather low average lap shear strength, and a generally decreasing lap shear strength with increasing speed. Varying amounts of un-welded energy director were seen for all applied welding speeds, an example of which is provided in Figure 11, which shows the fracture surfaces of a sample welded with a speed of 4 mm/s and an amplitude of 50%. All welds performed fell into category III, in accordance with the formerly introduced classification.

**Figure 12:**
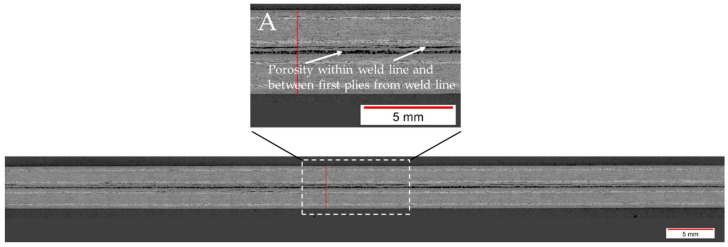
Microscopic cross-section of a sample welded with 100% amplitude and 32 mm/s welding speed using the thin energy director. Porosity is visible within the weld line and between the first and second plies of the adherends (Detail A).

**Figure 13:**
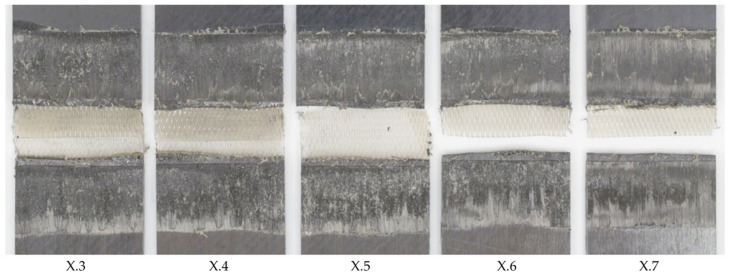
Fracture surface of sample welded with 100% amplitude and 24 mm/s welding speed using the thin energy director (samples X.3–X.7 from left to right). The fracture surface shows completely molten energy director but porosity within the weld line.

**Figure 14:**
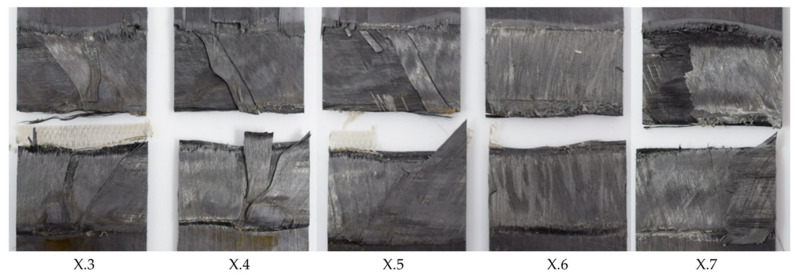
Fracture surface of sample welded with 100% amplitude and 16 mm/s welding speed using the thin energy director (samples X.3–X.7 from left to right). The fracture surface shows failure mostly in the 135° ply and fibre misalignment.

For the 100% amplitude case, significant scatter and an overall lower average lap shear strength compared to the baseline case were observed (Figure 10). All welds obtained at welding speeds of 22 mm/s or higher showed varying porosity in the weld area. A representative example of this is shown in Figure 12. The microscopic cross-section shows porosity within the weld line and between the first and second plies from the weld line. The effect was consistent for the whole weld line and the different welding speeds. Figure 13 shows the fracture surface of a sample welded with a welding speed of 24 mm/s. Porosity is clearly visible in the weld area, but no un-welded energy director was found. Although significant porosity is found in the weld lines, all welds performed using welding speeds between 20 and 32 mm/s fall into category II welds, as no un-molten energy director is present. The welds performed with welding speeds of 34 mm/s and above show different amounts of un-molten energy director and fall into category III. Alternatively, Figure 14 shows the fracture surface of a sample welded with 100% amplitude and a speed of 16 mm/s. The fracture surfaces showed a similar effect to that described for samples welded with low speeds and an initial vibration amplitude of 75%, falling into category I welds. Fibre squeeze was visible and led to a change in the failure mode. Within Figure 13, it can even be observed that the fibres of the 135° layer were not straight anymore, as a certain waviness was visible.

### 3.4. Consolidation Time

**Figure 15:**
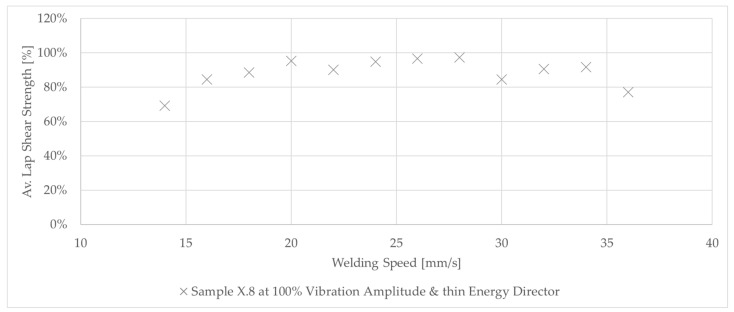
Graph shows the lap shear strength of the last (X.8) of the samples welded with 100% amplitude using the thin energy director at different welding speeds. The lap shear strength is similar for the majority of the applied welding speeds while only the lowest and highest speeds show a significant drop.

**Figure 16:**
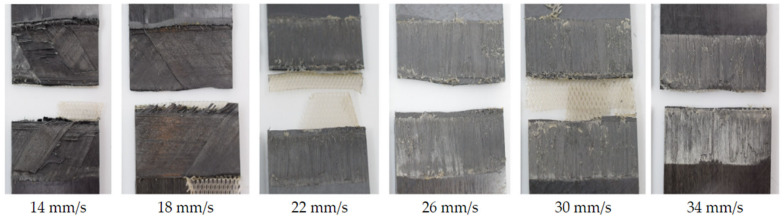
Fracture surfaces of samples which were subject to longer consolidation time. The samples were welded with 100% amplitude using the thin energy director and different welding speeds. The fracture surfaces show fibre squeeze out and areas of discoloured polymer matrix for welding speeds of 14 and 18 mm/s, while all fracture surfaces are free of porosity.

As described in Section 2, the Material and methods section, the X.8 samples (Figure 3) were considered separately as they were subjected to longer consolidation times. Figure 15 shows the lap shear strength of samples welded with 100% vibration amplitude and different welding speeds. The graph shows that, in all cases, the lap shear strengths were between 70% and 98% of that of the consolidated reference. Only samples with the lowest and highest welding speeds showed a rather significant drop in lap shear strength. The corresponding fracture surfaces are shown in Figure 16. Welds performed at speeds of 18 mm/s and below showed a different failure mode than the rest (second- or third-ply failure versus failure at the welding interface). Even areas of discoloured polymer matrix, indicating degradation, could be observed on those fracture surfaces. For high welding speeds (34 mm/s and above), some un-welded areas were visible, especially at the transversal edges. No porosity was visible on any of the fracture surfaces.

### 3.5. Energy Director

**Figure 17:**
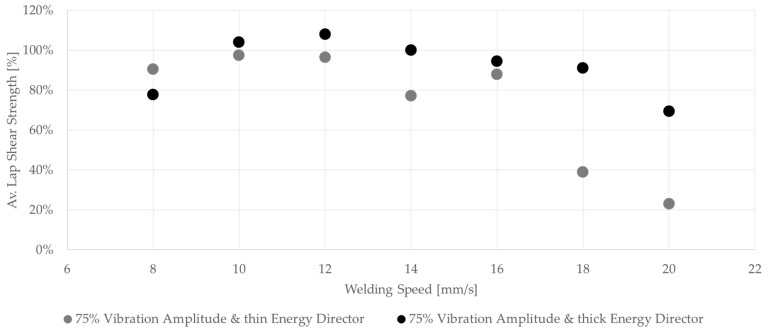
Graph compares the average lap shear strength of samples welded with 75% amplitude using the thin and the thick energy directors at different welding speeds. For the thick energy director, with higher speeds (up to 18 mm/s), welds with good welding quality can be achieved that fall into category II (error bars are excluded for readability and are shown separately in Figure 19).

**Figure 18:**
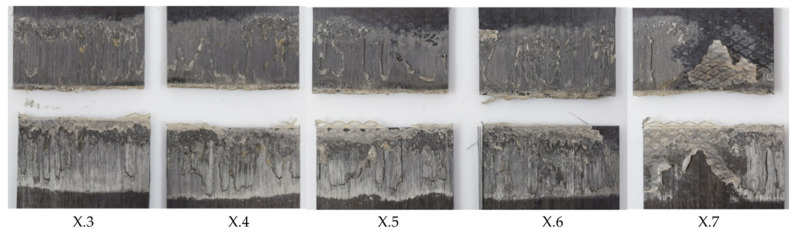
Fracture surface of sample welded with 75% amplitude and 20 mm/s welding speed using the thick energy director (samples X.3–X.7 from left to right). The fracture surface shows un-welded energy director, especially for the last sample.

Figure 17 shows the average lap shear strengths of samples welded at different speeds using the thin and thick energy directors. The achieved lap shear strengths showed a lot of similarities for welding speeds between 8 and 12 mm/s. This was also true for the visual aspect of the corresponding fracture surfaces. For speeds above 12 mm/s, the average lap shear strength for the thin energy director dropped significantly. In contrast, the average lap shear strength for the thick energy director welds showed only a slight decrease in average lap shear strength up to 18 mm/s. For welding speeds above 18 mm/s, the average lap shear strength was still somewhat higher compared to the thin energy director but, similarly, un-welded energy director was visible on the corresponding fracture surfaces (Figure 18).

## 4. Discussion

### 4.1. Welding Speed

From Figure 6 it is apparent that the welding speed has a significant influence on the weld quality. Compared to the baseline parameters, a decreased welding speed results in increased through-the-thickness heating due to the longer effective heating time. As a result, failure occurs within the 135° ply of the adherends instead of the welding interface (Figure 6). This is believed to be the consequence of fibre and polymer squeeze flow and resulting significant fibre distortion in several layers of the adherends. Within our previous study [15], we had already observed the sensitivity of unidirectional fibre arrangements to transverse squeeze flow compared to fabrics, which have an intrinsic stability owing to their textile architecture. A similar behaviour can be expected for continuous welding. It is also known that these effects do not necessarily affect the lap shear strength [7,15], as the complex stress state does not seem to require perfectly aligned fibre architectures to achieve a high lap shear strength, which can explain the only moderate drop in lap shear strength compared to the baseline weld parameters (Figure 1). 

An increased welding speed, on the other hand, has a more significant effect on the resulting lap shear strength. This is consistent with the appearance of un-welded and resin-rich areas on the fracture surfaces, as shown in Figure 8. The un-welded areas are a result of an insufficient time for the melting of the energy director. The occasional lack of contact between the energy director and adherends observed in microscopic cross-sections (Figure 9) confirms the existence of such un-welded areas. Resin-rich areas in the weld interface and, therefore, a thicker weld line are also a result of short heating times, since they lead to lower maximum temperatures in the weld line and, hence, reduced polymer flow and squeeze out upon application of the consolidation pressure.

If the welding speeds are only changed slightly compared to the baseline parameters (e.g., in the range of 10–12 mm/s), the results are very similar when comparing the lap shear strengths and the corresponding fracture surfaces (Figure 6). As already shown in previous research on the continuous ultrasonic welding of fabric-reinforced composites [7], this range of speeds is very small, which indicates that the process is very sensitive to changes in the welding speed. Similarly, in static ultrasonic welding, the welding stage where the highest mechanical performance is observed can be usually achieved within a narrow interval of welding times [10]. As continuous ultrasonic welding is performed with a constant welding speed, the welding speed is comparable to the weld time in static ultrasonic welding. The relation between the welding speed and welding time can be expressed by the following equation, using the welding time (*t_w_*), the welding speed (*v_w_*), and the width of the sonotrode (*W_s_*):

(2) $$v_{w}\times W_{s}=t_{W}$$

### 4.2. Amplitude

From current knowledge on the static ultrasonic welding processes, higher amplitudes can be expected to require lower welding speeds and lower amplitudes to require higher weld speeds in order to achieve similar weld quality [10]. This is due to decreased or increased overall heating rates caused by a decrease or increase in the vibration amplitude, respectively. With regards to the effect of the welding speed at different levels of vibration amplitude, a similar behaviour as that shown in Figure 6 (increasing-decreasing trend for the weld strength as the welding speed increases, with a narrow maximum weld strength plateau in between) is nevertheless expected, although shifted towards higher or lower welding speeds for increased or decreased amplitudes, respectively. In the case of higher and lower amplitudes, the trends were different to what has been expected.

The overall low lap shear strength in the low-amplitude case is consistent with the significant amount of un-welded energy director observed on the fracture surfaces (see Figure 11) and can be explained by the overall lower heating rates and is in line with previous research. Even for the lowest applied welding speed, the resulting weld time is not sufficient to achieve complete melting of the energy director. This is consistent with the behaviour observed in the baseline case at higher welding speeds (category III in Figure 6).

The evolution of the lap shear strength in the high-amplitude case shows a trend that significantly differs from that of the baseline case. Indeed, the highest average lap shear strength was obtained at the lowest welding speed tested in that case despite the fact that the fracture surfaces of those welds show significant fibre misalignment (Figure 14) and even polymer discoloration, which is interpreted as a sign of polymer degradation due to overheating (Figure 16). It is known from the literature that the onset of degradation does not necessarily lead to the immediate loss of properties and can even be beneficial due to cross-linking, which could explain the highest lap shear strength being observed at the lowest welding speed even with some signs of degradation [16]. The subsequent relatively low lap shear strength values in combination with high scatter (Figure 10), together with the presence of significant porosity in the weld lines (Figure 12) and fracture surfaces (Figure 13), is attributed to insufficient consolidation. The consolidation times for those welding speed range from 2 to 3 s. The reader should note that the length of the consolidation unit was kept constant in this study. It is thus reasonable to think that, for the higher welding speeds, as tested in the high-amplitude case, the consolidation unit might not have been long enough to ensure a sufficient consolidation time. From the literature it is known that a consolidation time of 3 s is not sufficient. Jongbloed et al. [17] showed that the best results can be obtained with a consolidation time of ca. 3.5 s with the welding parameters used in their study (35 mm/s welding speed). This hypothesis was confirmed by the fact that both the lap shear strength (Figure 15) and the fracture surfaces (Figure 16) of the last sample of each weld, which, due to the design of the welding process, were consolidated for 8 s, showed similar values and features, respectively, as the baseline case. It has to be noted that a longer consolidation unit poses challenges for the welding process if it is applied to actual parts, as it increases the footprint of the welding end-effector and can limit accessibility in actual production environments. Therefore, a smaller consolidation unit would be preferred.

Significant scatter is observed for the high-amplitude case, which can be explained by a lack of consolidation. Also, in the low-amplitude case and the baseline amplitude case (75%), significant scatter in the weld quality can be observed, especially for higher weld speeds. Similarly to observations in static ultrasonic welding and based on the fracture surfaces of those welds (Figure 8 and Figure 11), the scatter is not caused by insufficient consolidation but by the presence of resin-rich areas and un-welded energy director. This effect can be explained by the way the energy director melts. The melting of the energy director happens through the nucleation and growth of hot spots [9]. These two mechanisms depend on the welding parameters. Low amplitudes decrease the overall heating rates and, in combination with heat transfer into the adherends, limit the growth of hot spots. Consequently, this causes the process to rely on the nucleation of new hot spots, which can be subject to more randomness as it depends on intimate contact between the energy director and the adherends [9]. This can result in larger scatter between different welds using the same parameters. This effect becomes more pronounced with the relatively low welding force used in this investigation, as it reduces the intimate contact, which is a main driver for the nucleation of hot spots. In static welding, the varying weld times for lower amplitudes are compensated for by using vertical displacement to control the process. Comparable to weld-time-controlled welding in the static process, in continuous welding, a constant welding speed is usually applied.

### 4.3. Energy Director

**Figure 19:**
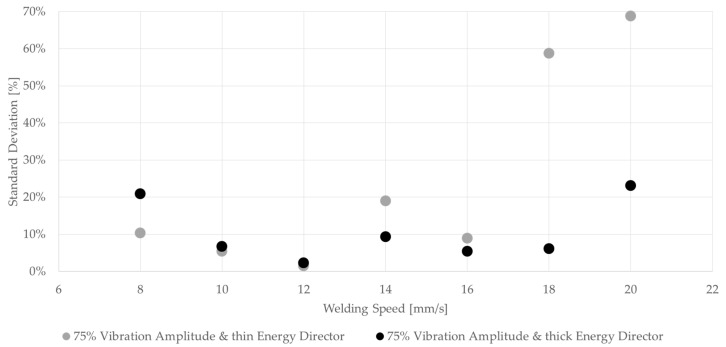
Comparison of standard deviation between thin and thick energy directors using 75% vibration amplitude. Standard deviation is shown as percentage of average lap shear strength.

A thicker energy director leads to similar trends as the baseline case but with a wider speed range, leading to sufficient weld quality without signs of un-welded energy director as the welding speed increases. An increased energy director thickness affects the compliance of the energy director itself. Derived from previous work [7], the compliance of the energy director, *C*, can be calculated based on the following formula:

(3) $$C=\frac{t}{A\times E}$$

where *t* is the thickness of the energy director, *E* the compressive modulus of the material, and *A* the area of contact between the energy director and the adherends. Since the material of the energy director and its effective area of contact remain the same for the two types of energy director used in this study, its compliance is directly related to its thickness (*t*). Hence, the thick energy director used within this study has a 2.5-times higher compliance compared to the baseline energy director. A more compliant energy director results in more uniform contact between the energy director and the adherends, and, therefore, to more nucleation of hot spots as described in the previous sub-section [7], which is believed to result in more uniform melting of the energy director. More uniform melting of the energy director results in a reduced amount of un-welded energy director, and, therefore, can be a major reason for the overall higher average lap shear strength (Figure 17), as well as less scatter within one weld line. When comparing the standard deviations as percentages of the average lap shear strength, as shown in Figure 19, this effect is mostly apparent for higher welding speeds. As described earlier in the case of the baseline energy director, the appearances of un-welded energy director and resin-rich areas differ significantly from sample to sample, causing the high standard deviation we can observe in Figure 19. Contrarily, the standard deviation for the thicker energy director case remains rather low even for higher welding speeds. An exception has to be made for the 20 mm/s and the 8 mm/s cases. For the 20 mm/s case, a potentially insufficient consolidation time and the resulting porosity as well as un-welded energy director (Figure 18) play a role in increasing the scatter. In the case of the 8 mm/s welding speed, fibre misalignment due to through-the-thickness heating and a change in failure mode were observed, which might lead to higher scatter in lap shear strength results

Comparing the welding speeds of the baseline and the thick energy director case, similar or even higher welding speeds for the thick energy director case lead to category II welds. Contrarily, from the literature, it is expected that a thicker energy director results in longer weld times owing to lower heat generation [8], and should therefore also require slower welding speeds in the continuous process, but we cannot see this in the results of the welding trials. From research on static welding, it is known that the differences are rather small. In the case of doubling the energy director thickness from 0.25 mm to 0.5 mm, an average weld time increase of only 10% was found [8]. We believe that the improved uniformity in the melting of the energy due to its higher compliance overshadows the effect of reduced heating rates and, therefore, the expected lower welding speeds for the thicker energy director cannot be observed from the results.

## 5. Conclusions

This paper investigated the influence and interdependence of the welding speed, amplitude, and energy director thickness on the weld quality when adherends made of unidirectional materials and a robotic continuous ultrasonic welding process were used. 

We conclude that the continuous welding process is highly affected by changing welding speeds if vibration amplitudes of 75% (of the maximum vibration amplitude) are used. Small changes in the welding speed can lead to significant changes in the weld quality along the weld area, which leads to fibre distortion (for low welding speeds) and un-welded areas (for high welding speeds).

Lower amplitudes reduce the overall heating rates and require longer weld times, which cannot be achieved with feasible welding speeds. This results in un-welded areas even for the lowest possible speed. Lower amplitudes lead to significant scatter in the weld quality, which can be explained by the nucleation of hot spots, instead of the growth of hot spots, being the dominant mechanism.

Higher vibration amplitudes increase the heating rates and lead to fully molten energy director even for higher welding speeds. But insufficient consolidation results in porosity within the weld line, which can be mitigated by a longer consolidation unit but can pose challenges in the method’s actual application in production environments.

Thicker and, therefore, more compliant energy directors lead to more uniform melting of the energy director and therefore less deviation in the weld quality and lap shear strength, making the process less sensitive to changing welding speeds. Despite the expected lower heating rates for thicker energy directors, similar welding speeds can be used to achieve complete melting of the energy director.

## Figures and Tables

**Table 1:** Test matrix showing the combinations of process parameters applied during the study.

Vibration Amplitude [%]	Energy Director Areal Weight (g/m^2^)	Welding Speed [mm/s]
75	160	8, 10, 12, 14, 16, 18, 20
50	160	4, 6, 8
100	160	14, 16, 18, 20, 22, 24, 26, 28, 30, 32, 34, 36
75	400	8, 10, 12, 14, 16, 18, 20

## Data Availability

Data are contained within the article.
